# Validation and Reliability of a Classification Method to Measure the Time Spent Performing Different Activities

**DOI:** 10.1371/journal.pone.0128299

**Published:** 2015-06-08

**Authors:** Marie-Ève Riou, François Rioux, Gilles Lamothe, Éric Doucet

**Affiliations:** 1 Behavioural and Metabolic Research Unit (BMRU), School of Human Kinetics, Faculty of Health Sciences, University of Ottawa, Ottawa, Canada; 2 Frisoft Inc., Gatineau, Canada; 3 Department of Mathematics and Statistics, Faculty of Science, University of Ottawa, Ottawa, Canada; The University of Queensland, AUSTRALIA

## Abstract

The aim of this study was to validate the performance and reliability of results obtained from a classification model that measures time spent performing activities in confined (CE) and unrestricted (UE) environments. In CE, participants wore a pair of biaxial and/or triaxial accelerometers while performing pre-determined training activities classified as variants of lying down, dynamic standing, sitting, walking and running on two separate days. A classification model trained with activities performed in a specific order during the first day was developed to validate the activities performed in a random order on the second day (CE) and over 24 hours on a separate day (UE). The performance of the classification model was validated against triaxial accelerometers using six (x, y and step counts for arm and thigh) or eight (same as six features plus z axis) features. The reliability of the classification model was tested in both environments using six features. Results revealed an overall accuracy of 94% in CE and 90% in UE. The sensitivity in CE and UE was 94% and 95% for lying down, 88% and 80% for dynamic standing, 97% and 89% for sitting, 96% and 78% for walking and 90% and 64% for running, respectively. No significant differences were noted between performances obtained with six or eight features. Results were highly reproducible in both environments. The results obtained from the classification model were accurate and reproducible, and highlight the potential use of this approach in research to quantify the time spent performing different activities.

## Introduction

Activity monitoring systems are used to estimate energy expenditure using data captured by accelerometers and other sensors. They have been widely used due to their small size, low cost, and low power consumption [1, 2]. Nevertheless, the measurement of energy expenditure does not allow the characterization of the different activities performed during a determined time frame. As reviewed by Preece *et al* (2009) and Yang & Hsu (2010), activities performed can be computed from raw accelerometry data using classification models that are obtained from machine learning classifiers (*e*.*g*., decision trees, neural networks, Bayesian classifiers, support vector machines and others).

The validation of classification models aimed at recognizing activities has been conducted in a confined environment (CE), showing a high accuracy [3–6]. Some studies have also been performed in a semi-unsupervised environment showing similar results [7–13]. Under a free unsupervised period of 4 hours, Ermes [14] showed a sensitivity (chances of classifying an activity as positive when it is indeed positive) of 98% for lying down, 80% for sitting/standing and 30% for walking when four annotated activities were considered over a total of nine recognized activities. Long *et al*. also demonstrated a sensitivity of 80% for walking and 93% for running when participants annotated five activities over a 10-hour period when using one accelerometer [15]. However, to our knowledge, no study has validated the results obtained from a classification model over 24 hours in an unrestricted environment (UE). One of the reasons is that the internal memory of devices is limited in size and quickly fills up when data are sampled at a high frequency. Nevertheless, recognizing only major categories of activities (*i*.*e*., lying down, dynamic standing, sitting and walking) does not require a high data-sampling frequency due to the nature of these activities and thus makes the recording over a longer period of time possible.

The first objective of this study was to build a classification model for biaxial and triaxial accelerometers and to validate the performance of this classification model in discriminating five different activities. Specifically, the performance was validated using data gathered under 2 hours in CE and under 24 hours in UE. The second objective was to compare the performance of the classification model using a set of six (x, y and step counts for arm and thigh) or eight (x, y, z and step counts for arm and thigh) features recorded from the triaxial accelerometers. The third objective was to assess the reliability of the results acquired from the classification model obtained with biaxial and triaxial using six features under CE and UE. We hypothesized that (a) the results would be highly accurate in both environments using biaxial or triaxial accelerometers (b) the performance of the classification model obtained with six or eight features would be similar and (c) the results obtained from the classification model would be highly reproducible in both the CE and UE.

## Materials and Methods

### Participants

A total of seventeen males and nineteen females students were recruited to complete this set of experiments. The inclusion criteria were as follows (a) over the age of 18 years; (b) stable weight (±2 kg) within the past six months; (c) nonsmokers; (d) no drug or alcohol abuse; and (e) without any orthopedic limitation. All experiments were conducted according to the guidelines laid down in the Declaration of Helsinki and all the procedures involving human participants were approved by the University of Ottawa ethics committees. Written informed consent was obtained from all participants.

### Accelerometers

A pair of biaxial and/or triaxial activity-monitoring systems (accelerometers) (SenseWear Pro 3 Armbands, HealthWear Bodymedia, Pittsburgh, PA) were used. SenseWear Pro 3 Armbands were chosen because they provide access to raw data (acceleration axes and step counts) and provide accurate estimates of energy expenditure [16]. One accelerometer was placed around the upper arm (midway between the acromion and the olecranon) while the other was placed around the thigh (midway between the patella and the inguinal fold; on the exterior of thigh). The internal clocks of both accelerometers were synchronized before the beginning of each session with the researcher’s watch or with the participants’ watch. The data recorded over time were the following features: x and y acceleration axes and the step counts (for the arm and thigh) while using a biaxial accelerometer and the x, y and z acceleration axes and the step counts (for the arm and thigh) while using a triaxial accelerometers. Therefore, the biaxial accelerometers provided six features while the triaxial accelerometers provided eight features. In terms of anatomic axes, the x, y and z axes represent the horizontal, vertical/frontal and sagittal axes, respectively. The acceleration measures were accumulated and averaged over a period of 5s while the step count measures are accumulated and averaged over a one minute interval. The combination of the accelerations and step counts recorded every five seconds refer to one data sample. Note that the step counts measure was stable during one minute while the accelerations were different every five seconds.

### General Procedures of the Study

**Fig 1:**
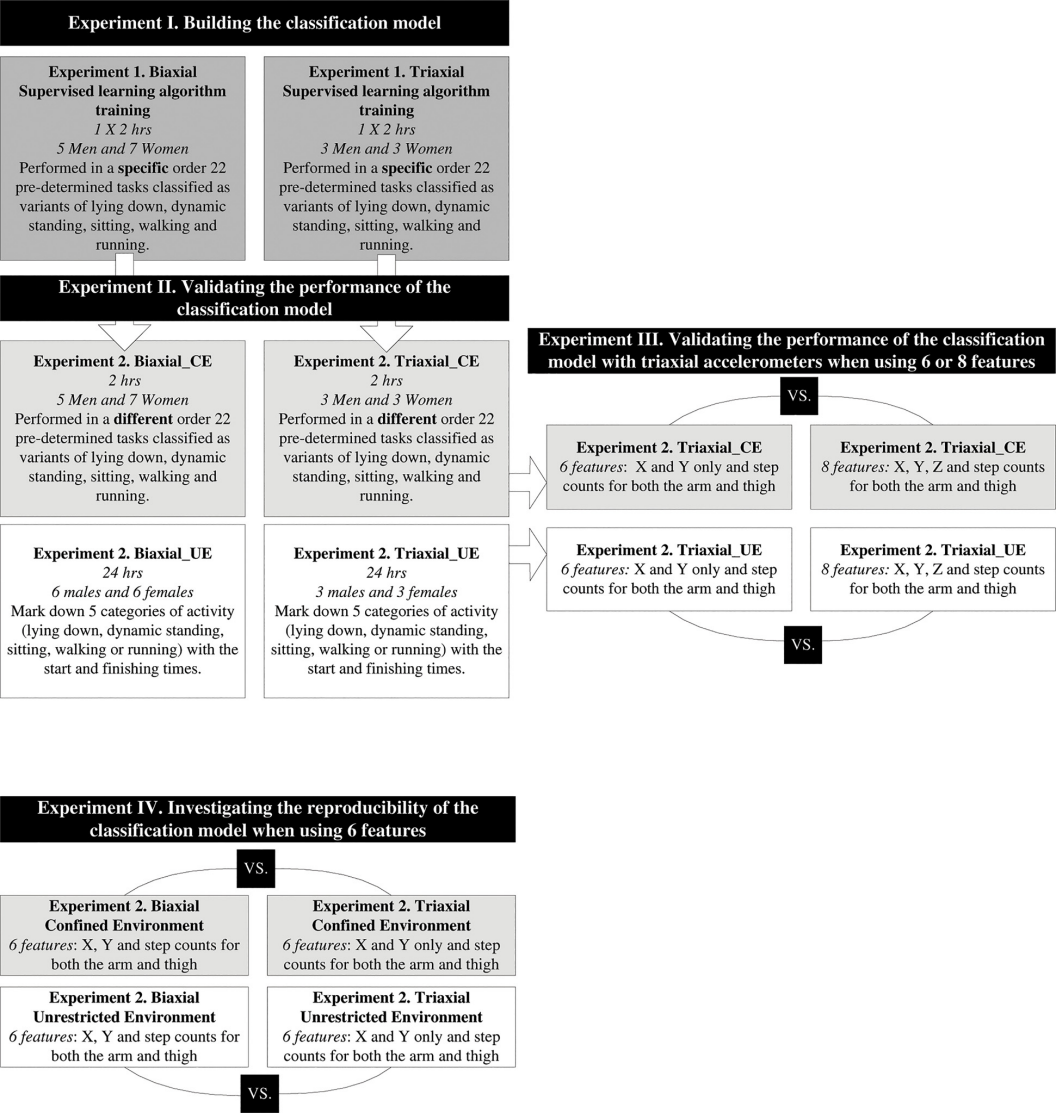
General Procedures of the Study.

This study consisted of four experiments (Fig 1) (a) Building the classification model with biaxial and triaxial accelerometers (Experiment I); (b) Validating the performance of the classification model in CE and in UE with biaxial and triaxial accelerometers (Experiment II); (c) Validating the performance of the classification model with triaxial accelerometer when using six or eight features (Experiment III); (d) Investigating the reliability of results obtained from the classification model when using six features recorded by a biaxial and a triaxial accelerometers under CE and UE (Experiment IV). The four experiments are further described in the following sections.

#### Experiment I—building the Classification Model

The data (acceleration axes and step counts) were obtained from accelerometers worn by participants performing 22 predetermined training activities classified as variants of lying down, dynamic standing, sitting, walking, running, biking, and climbing stairs in a specific order. The procedures were performed under the supervision of the researcher who recorded the beginning and end of each activity.

**Fig 2:**
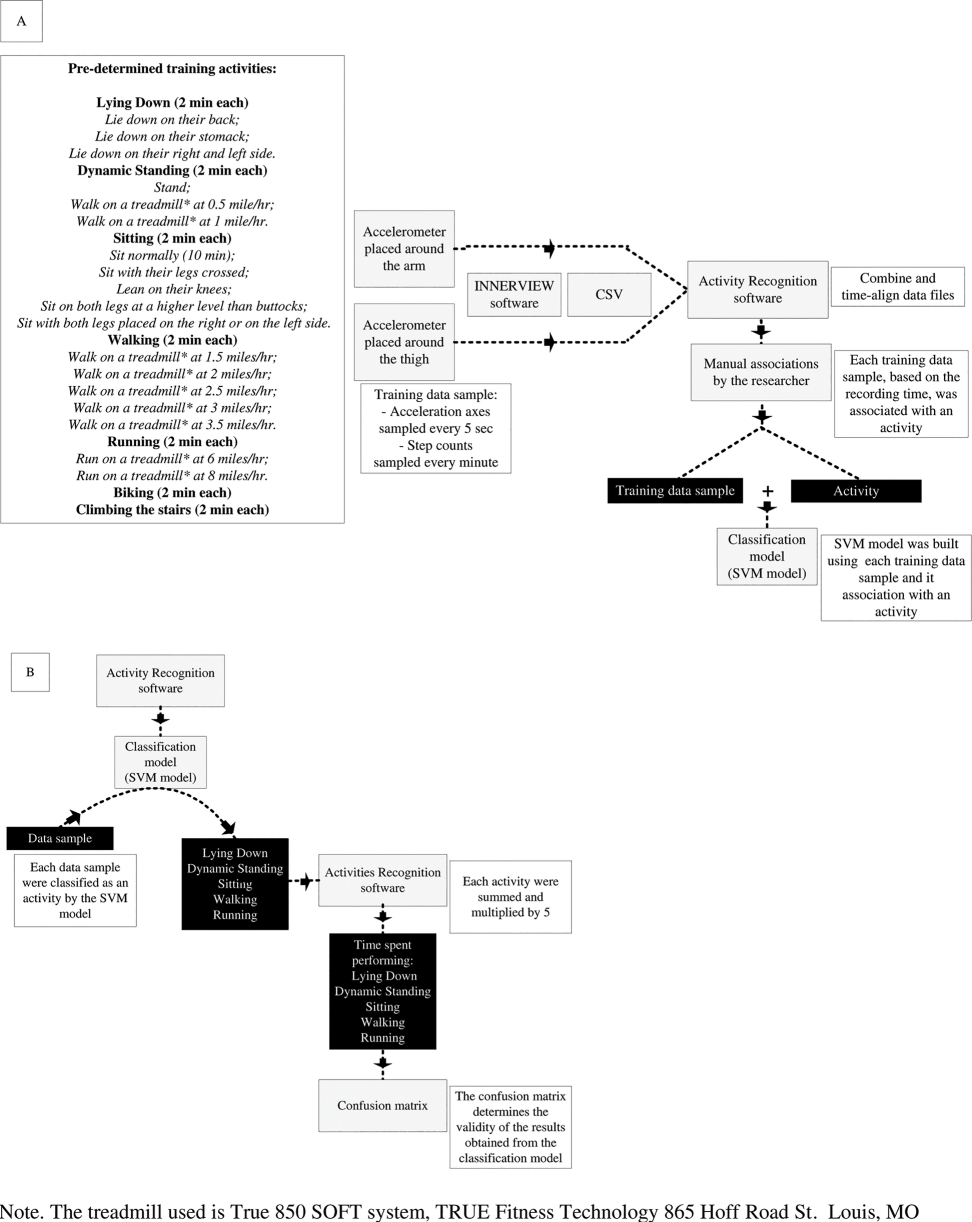
Building the Classification Model (A) and Obtainment of the Time Spent Performing Activities (B).

The INNERVIEW software (version 4.02; Bodymedia, Pittsburgh, PA) was used to extract the data obtained from the 22 predetermined training activities (training data) from the accelerometers (Fig 2A). Training data were exported in two Comma-Separated Values (CSV) files: one file for the accelerometer worn on the arm and one for the accelerometer worn on the thigh. *Activity Recognition* software was used to combine and to synchronize these two training data files, which produced a single file containing a sequence of training data samples. The associated activity for each sample was then identified based on the recording time. Transitions from one activity to another were manually removed from the training data set. Two classification models (support vector machines, kernel type: radial basis function; cost: 10; gamma parameter: 0.01) were then built using those training samples (recorded features and known activity): one classification model with the biaxial accelerometers (*Experiment 1*: *Biaxial*) and one with the triaxial accelerometers (*Experiment 1*: *Triaxial*). The *Activity Recognition* software uses the SVM implementation of the open source software library Java-ML as a classification algorithm [17]. To facilitate the discrimination between variants of dynamic standing and walking, a threshold of 30 steps per minute or less was used. The threshold was applied during data pre-processing in the training phase. If the step count for one data sample was lower than 30 steps per minute, it was assigned a value of 0 steps per seconds, and then fed to the classifier as a training sample. The rationale for using a 30 step counts per minute threshold is based on the reasoning that dynamic standing could be associated with minor lower body movement at low speeds for short distances (which is equivalent to one step every two second or less).

#### Experiment II—validating the performance of the Classification Model

To validate the performance of the classification model under CE (*Experiment 2*: *Biaxial_CE and Experiment 2*: *Triaxial_CE*), participants were asked to perform the same 22 predetermined training activities in a different order, which was different for each participant. After initial analyses, low accuracy of biking and climbing stairs (*i*.*e*., 37% for climbing the stairs and 74% for biking in *Experiment 2*: *Biaxial_CE*) were obtained. Therefore, these activities were removed from the classification models and were not further classified as part of this study. The classification model therefore classified climbing stairs or biking as either walking or running. During the validation of the UE (*Experiment 2*: *Biaxial_UE and experiment 2*: *Triaxial_UE*), participants were asked to mark down on a sheet of paper five categories of activities (lying down, dynamic standing, sitting, walking or running) with the start and finish times (precision within one second) over a 24-hour period. Dynamic standing was described to the participants as a static standing position that could include dynamic movement of the upper body. Since it could be associated with minor lower body movement, walking for short distances (less than 30 steps per minute) was also considered as dynamic standing. Examples include meal preparation, washing dishes, talking to someone while standing, etc. Walking was categorized as a displacement of more than 30 consecutive steps per minute. Examples include walking to work, walking to the bus stop, walking the dog, etc. Each data sample from *Experiment II* was obtained as previously described (*i*.*e*., INNERVIEW software, CSV, *Activities Recognition Software*) and classified as an activity either by the biaxial or triaxial classification model (Fig 2B). When participants were doing other types of activities, the latter were classified as one of the selected activities. The Activity *Recognition Software* was used to coordinate this sequence. The total time spent performing each activity was determined as the product of the sampling rate (5 s) and the number of occurrences of the different activities. The classification was then compiled in a confusion matrix to determine the validity of the results obtained from the classification model. Under UE, participants were instructed to remove the accelerometer during all water activity, including bathing, but to wear it overnight.

#### Experiment III—validating the performance of the Classification Model with triaxial accelerometer when using six or eight features

For this experiment, data samples obtained from six (*i*.*e*., x, y axes and step counts times two accelerometers) and eight features (*i*.*e*., x, y and z axes and step counts times two accelerometers) were compared in a CE and in a UE. The results in terms of activity classification were obtained from the same triaxial accelerometers while either including (eight features) or removing the z axis (six features) (Fig 1).

#### Experiment IV—investigating the reliability of the results obtained from the Classification Model using six features

The reliability of the results obtained from the classification model using six features was investigated. Results of *Experiment 2*: *Biaxial* were compared with results of *Experiment 2*: Triaxial in both CE and UE (Fig 1). The z axis (arm and thigh) from each data sample obtained with the triaxial accelerometers was removed for this analysis.

### Statistical Analysis

Statistical analyses were performed in Excel (version 2007). Performance of the classification model was determined with the overall accuracy (*i*.*e*., mean proportion of all activities that are correctly classified per person) and using five indicators: sensitivity (chances of classifying an activity as positive when it is indeed positive), the positive predictive value (chances that an activity is indeed positive, when it is classified as positive), the F-Score (the "harmonic mean between sensitivity and positive predictive values" [18] $\left(2\frac{\left(sensitivity\cdot precision\right)}{\left(sensitivity+precision\right)}\right)$) and the specificity (chances of classifying an activity as negative when they are truly negative) in a confusion matrix. Cohen's kappa coefficient (measure of the agreement between the real activity and the classifications) was also determined [19]. In order to investigate the difference between the performance of the classification model using six or eight features, a Wilcoxon matched-pairs signed rank test was performed using statistical software (Prism v5, GraphPad Software Inc., San Diego, CA). To investigate the reliability of the results obtained from the classification model when using six features (overall accuracies) in CE and UE, an independent samples t-test was performed. The underlying assumption of normality of the two samples t-test was verified with a normal probability plot performed with Minitab 16. A linear tendency was observed in both plots suggesting that it is reasonable to assume that the accuracy is normally distributed (data not shown). However, since the slopes were very different in CE, the equality of the variance was not assumed. As a result, a t-test with a Welch correction was performed with the GraphPad Prism. Values are presented as percentage ± standard deviation.

## Results

The participants’ characteristics are described in **Table 1**.

**Table 1:** Participants Characteristics.

Experiment		Weight (kg)	BMI (kg/m^2^)	Age (yr)
***Experiment 2*. *Biaxial_CE*** ^a^ ^,^ ^b^ ^,^ ^c^	**All (N = 12)**	67.9 ± 11.2	23.5 ± 2.4	24.6 ± 4.6
	**Females (n = 7)**	60.2 ± 6.8	22.7 ± 1.8	24.7 ± 5.9
	**Males (n = 5)**	78.7 ± 4.8	24.5 ± 3.0	24.4 ± 2.4
***Experiment 2*. *Biaxial_UE***	**All (N = 12)**	68.0±12.4	23.6±3.0	26.7±2.9
	**Females (n = 6)**	58.9±6.3	22.4±1.1	26.0±2.8
	**Males (n = 6)**	77.2±10.1	24.9±3.9	27.3±3.1
***Experiment 2*. *Triaxial_CE*** ^a^ ^,^ ^d^	**All (N = 6)**	68.5±13.2	23.2±2.8	21.3±2.7
	**Females (n = 3)**	58.1±7.5	21.6±1.5	22.7±3.5
	**Males (n = 3)**	79.0±7.0	24.8±3.2	20.0±1.0
***Experiment 2*. *Triaxial_UE***	**All (N = 6)**	65.2±11.5	22.5±2.2	21.5±1.5
	**Females (n = 3)**	55.2±4.8	21.1±0.9	21.0±2.0
	**Males (n = 3)**	75.1±3.4	24.0±2.3	22.0±1.0

Values are mean ± SD; BMI = body mass index.

^a^ The same subjects for Experiment 1 and 2 were used.

^b^ A total of 13 participants, were recruited. However, one participant had to be excluded because of incomplete accelerometry data.

^c^ For experiment 2 performed with Biaxial, three participants (2 male and 1 female) performed both the Biaxial_CE and the Biaxial_UE. Other subjects were different.

^d^ For experiment 2 performed with Triaxial, three participants (2 female and 1 male) performed both the Triaxial_CE and the Triaxial_UE. Other subjects were different.

### Phase II—Validating the Performance of the Classification Model in CE

The confusion matrix presented in **Table 2**shows the real and classified time (s) spent performing activities in CE. The classification model had an overall accuracy of 94±4%, including lying down, dynamic standing, sitting, walking and running. The sensitivity was higher than 90% for all the time spent in activities except for dynamic standing, which had the lowest classification results (88±18%). The positive predictive value was 95±8% for lying down, 95±8% for dynamic standing, 98±3% for sitting, 66±8% for walking, and 88±18% for running. The F-score demonstrated a high overall performance for lying down, dynamic standing, sitting, running, and with the lowest value for walking (76±16%). The high specificity (higher than 99% for most of the activities) suggested that the classification model can accurately detect a specific activity with limited false-positive values. Finally, the association between the real activities and the classification, measured with Cohen’s Kappa Coefficient, indicated that the classification model developed in CE highly agrees with the reality (0.93±0.004).

**Table 2:** Confusion Matrix of the Time Spent Performing Activities Obtain from the Results of the Classification Model in CE Sessions.

		**Class**					
		**Walking**	**Sitting**	**Running**	**Lying down**	**Dynamic Standing**	**Overall**
**Real**	**Walking**	**1334**	0	36	0	24	
	**Sitting**	0	**2552**	0	55	10	
	**Running**	60	0	**478**	0	0	
	**Lying down**	9	43	0	**1057**	15	
	**Dynamic Standing**	96	9	0	0	**734**	
**Overall Accuracy (%)** ^**a**^							94±4
**Sensitivity (%)** ^**b**^		96±12	97±4	90±20	94±7	88±18	
**F-Score (%)** ^**c**^		76±16	98±2	88±18	94±6	90±11	
**Specificity (%)** ^**d**^		88±4	99±2	99±2	99±2	99±1	
**Kappa** ^**e**^							0.93±0.00
**Linear Weight Kappa**							0.90±0.01
**Quadratic Weighted Kappa**							0.87±0.01
		**Class**					
		**Walking**	**Sitting**	**Running**	**Lying down**	**Dynamic Standing**	**Overall**
**Real**	**Walking**	**1334**	0	36	0	24	
	**Sitting**	0	**2552**	0	55	10	
	**Running**	60	0	**478**	0	0	
	**Lying down**	9	43	0	**1057**	15	
	**Dynamic Standing**	96	9	0	0	**734**	
**Overall Accuracy (%)** ^**a**^							94±4
**Sensitivity (%)** ^**b**^		96±12	97±4	90±20	94±7	88±18	
**F-Score (%)** ^**c**^		76±16	98±2	88±18	94±6	90±11	
**Specificity (%)** ^**d**^		88±4	99±2	99±2	99±2	99±1	
**Kappa** ^**e**^							0.93±0.00
**Linear Weight Kappa**							0.90±0.01
**Quadratic Weighted Kappa**							0.87±0.01

^a^ Overall accuracy is the mean proportion of all activities that are correctly classified per person

^b^ Sensitivity corresponds to the chances of classifying an activity as positive when it is indeed positive

^c^ F-Score is defined as the "harmonic mean between sensitivity and positive predictive values" [18]

^d^ Specificity is a measure of chances of classifying an activity as negative when they are truly negative

^e^ Cohen’s Kappa is the measure of the agreement between the real activity and the classifications

### Phase II—Validating the Performance of the Classification Model in UE

The confusion matrix presented in **Table 3**presents the real and classified time (s) spent performing activities in UE. The classification model had an overall accuracy of 90±4% and a sensitivity that varies between 64 and 95%. Of all activities, lying down and sitting had the highest sensitivity. The positive predictive values were 85±9% for lying down, 76±12% for dynamic standing, 85±6% for sitting, 56±21% for walking and 88±18% for running. Since walking had the lowest sensitivity and positive predictive value, it had an F-Score of 62±18%. The high specificity (between 87 and 100%) and a Cohen’s Kappa Coefficient of 0.85±0.001 suggested respectively that the classification model had a low false-positive rate and that there was a high degree of agreement between the reality and the classification.

**Table 3:** Confusion Matrix of the Time Spent Performing Activities Obtained from the Results of the Classification Model in UE Sessions.

		**Class**					
		**Walking**	**Sitting**	**Running**	**Lying down**	**Dynamic Standing**	**Overall**
**Real**	**Walking**	**7663**	318	60	17	1471	
	**Sitting**	515	**80640**	0	6495	2307	
	**Running**	260	0	**780**	0	0	
	**Lying down**	22	3855	0	**69878**	89	
	**Dynamic Standing**	2418	1454	0	266	**19937**	
**Overall Accuracy (%)** ^**a**^							90±4
**Sensitivity (%)** ^**b**^		78±16	89±11	64±27^**f**^	95±4	80±7	
**F-Score (%)** ^**c**^		62±18	83±7	73±24^**f**^	86±4	75±8	
**Specificity (%)** ^**d**^		90±2	88±4	100±0^**f**^	87±6	90±2	
**Kappa** ^**e**^							0.85±0.00
**Linear Weight Kappa**							0.81±0.00
**Quadratic Weighted Kappa**							0.77±0.00
		**Class**					
		**Walking**	**Sitting**	**Running**	**Lying down**	**Dynamic Standing**	**Overall**
**Real**	**Walking**	**7663**	318	60	17	1471	
	**Sitting**	515	**80640**	0	6495	2307	
	**Running**	260	0	**780**	0	0	
	**Lying down**	22	3855	0	**69878**	89	
	**Dynamic Standing**	2418	1454	0	266	**19937**	
**Overall Accuracy (%)** ^**a**^							90±4
**Sensitivity (%)** ^**b**^		78±16	89±11	64±27^**f**^	95±4	80±7	
**F-Score (%)** ^**c**^		62±18	83±7	73±24^**f**^	86±4	75±8	
**Specificity (%)** ^**d**^		90±2	88±4	100±0^**f**^	87±6	90±2	
**Kappa** ^**e**^							0.85±0.00
**Linear Weight Kappa**							0.81±0.00
**Quadratic Weighted Kappa**							0.77±0.00

^a^ Overall accuracy is the mean proportion of all activities that are correctly classified per person

^b^ Sensitivity corresponds to the chances of classifying an activity as positive when it is indeed positive

^c^ F-Score is defined as the "harmonic mean between sensitivity and positive predictive values" [18]

^d^ Specificity is a measure of chances of classifying an activity as negative when they are truly negative

^e^ Cohen’s Kappa is the measure of the agreement between the real activity and the classifications

^f^ Only three participants had practiced this activity.

### Phase III—Validating the Performance of the Classification Model with Triaxial Accelerometer when using six or eight Features

**Table 4**presents results of the performance of the triaxial accelerometer when using six or eight features in CE and UE. The difference between both overall accuracies revealed no significant difference in CE (p = 0.81) and UE (p = 1.0).

**Table 4:** Confusion Matrix of the Time Spent Performing Activities with Triaxial Accelerometers when using six and eight Features.

		CE		UE^f^	
		6 features	8 features	6 features	8 features
**Sensitivity (%)** ^**a**^	**Walking**	91±7	91±7	83±6	83±6
	**Sitting**	92±8	94±7	93±3	94±5
	**Running**	64±25	64±25	-	-
	**Lying Down**	92±4	90±11	88±11	87±10^**A**^
	**Dynamic Standing**	85±11	87±9	79±8	79±8
**F-Score (%)** ^**b**^	**Walking**	85±6	85±6	77±4	77±4
	**Sitting**	94±5	95±3	90±5	90±5
	**Running**	69±21	69±21	-	-
	**Lying Down**	86±13	88±9	91±6	91±5^**A**^
	**Dynamic Standing**	88±6	89±5	83±5	84±5
**Specificity (%)** ^**c**^	**Walking**	94±3	94±3	98±1	98±1
	**Sitting**	98±1	97±2	90±5	89±7
	**Running**	99±2	99±2	100±0	100±0
	**Lying Down**	96±5	97±3	97±2	97±4
	**Dynamic Standing**	99±1	99±1	99±1	99±1
**Overall Accuracy (%)** ^**d**^		88±6	89±4	89±4	89±4
**Kappa** ^**e**^		0.84±0.01	0.85±0.00	0.82±0.00	0.83±0.00
**Kappa linear weight**		0.81±0.01	0.81±0.00	0.79±0.00	0.80±0.00
**Kappa quadratic weight**		0.79±0.02	0.79±0.02	0.76±0.01	0.76±0.01

^a^ Sensitivity corresponds to the chances of classifying an activity as positive when it is indeed positive

^b^ F-Score is defined as the "harmonic mean between sensitivity and positive predictive values" [18]

^c^ Specificity is a measure of chances of classifying an activity as negative when they are truly negative

^d^ Overall accuracy is the mean proportion of all activities that are correctly classified per person

^e^ Cohen’s Kappa is the measure of the agreement between the real activity and the classifications

^**f**^ In UE, the results for running are not included since no participants had practiced this activity and the results for lying down are based on 5 participants since one participant had not practiced this activity.

### Phase IV—Investigating the Reliability of the Results Obtained from the Classification Model using six Features

The analyses of the reliability of the results obtained from the classification model showed no significant differences for the overall accuracy in CE (p = 0.056) or UE (p = 0.447). The results confirmed with 95% confidence that the difference in the overall accuracies was 6.0% with a maximum error of 6.3% in CE. Similarly, the analyses revealed with 95% confidence that the difference in the overall accuracies was 1.6% with a maximum error of 4.3% in the UE.

## Discussion

To our knowledge, this is the first study to validate a classification model to determine the time spent performing activities in UE for a period of 24 hours. Collectively, these results indicate the relatively high performance of the classification model in CE and UE. Furthermore, the present findings demonstrate that including eight features *vs*. six features does not increase the performance of the classification model, at least when investigating the five categories of activities presented in this paper. Finally, the results obtained from the classification model showed a high level of reliability when using six features in both CE and UE.

### Phase II—Validating the Performance of the Classification Model in CE

The results obtained in CE suggest an overall accuracy of 94%. This accuracy is similar to that previously reported [4]. When further investigating the time spent in activities, our results showed a higher sensitivity than the one observed by Van Laerhoven in a case study measuring seven activities [5]. The only exception was for dynamic standing, which is 6% lower in our study [5]. The lower recognition accuracy for dynamic standing in our study could be related to the confusion involving the transition between the static and dynamic activities [20]. The classification model could not accurately recognize the transitions between each task, which highlights a need for machine-learning classifiers that can detect temporal sequences such as Hidden Markov Models. However, it could be speculated that in a normal environment, the number of transitions between different activities is relatively low compared to the transition that was done every 2 min in the CE protocol. Nevertheless, when dynamic standing was combined with walking, the sensitivity increased to 98±4%. The number of accelerometers used could also explain slight differences between studies. The classification model shows better accuracy for sitting and dynamic standing as did other studies that used multiple sensors [7, 9, 21] compared to studies that only used one accelerometer [4, 20, 22]. Our results as well as those from other studies [20, 23] emphasize the importance of using at least two sensors to improve the classification accuracy of sitting and dynamic standing. This is particularly important because these activities constitute a large proportion of daily activity in a modern environment [24].

### Phase II—Validating the Performance of the Classification Model in UE

Based on Foester’s research, a reduction in the overall accuracy of the classification model would have been expected in CE (95.8%) compared to UE (66.7%) (nine activities) [9]. The overall accuracy obtained from the 24 hours of participants’ annotations was only 4% lower, which is less than what has been observed by others [8, 14]. A closer inspection of our data revealed that the sensitivity for dynamic standing, walking, and running were the lowest. Nevertheless, after combining dynamic standing and walking, the sensitivity improved to 93±5%. These values are slightly better than those reported by Ermes *et al*. (2008) for 4 hours of testing when four out of nine activities were annotated by the participants. It is also important to note that the proportion of time was 37% or 8.9 hours for lying down, 12% or 2.9 hours for dynamic standing, 45% or 10.8 hours for sitting, 5% or 1.2 hours for walking and 0.05% or 0.01 hours for running. In this case, even if the proportion of time spent lying down and sitting (82% or 19.7 hours) is high, it represents the percentage of time spent in sedentary behaviors (*i*.*e*., lying down and sitting) generally observed in the population [24].

### Phase III—Validating the Performance of the Classification Model with Triaxial Accelerometer when using six or eight Features

It would seem logical that adding the z acceleration axes should lead to a better activity classification. However, this is not the case since no significant differences were noted between the overall accuracy when including or excluding the z axis of both accelerometers. It should be noted that the step count was part of both features sets. In addition, the activities analyzed were mostly performed in the x and y acceleration axes, which does make the inclusion of a third axis (z axis) rather unnecessary. We can thus conclude from our data that using a classification model that was trained using either six or eight features does not improve classification accuracy under the conditions described in this study.

### Phase IV—Investigating the Reliability of the Results Obtained from the Classification Model using six Features

Our results suggest that the classification model obtained in CE and in UE is reproducible. Indeed, the maximum error was.6.3% in CE and 4.3% in UE. A small difference between the internal clocks of both accelerometers and the researcher’s watch could have increased the variability across sessions in the CE. Similarly, the maximal error can be explained by the small difference between internal clocks of both accelerometers and the participant's watch in UE. The complexity and inconvenience related to the exact description of the movement second-by-second by the participant may have been associated to lower annotation compliance and thus may have lead to a certain degree of under-reporting that could have also reduced the reliability of the classification model.

### Limitations

Even if the classification model presented and discussed could be considered to have good classification accuracy in both CE and UE, several confounding factors should be considered and identified. Only 2 min in each activity were used to construct the classification model and the transition between the static and dynamic movements was not taken into account. In addition, even with pre-determined training activities classified as variants of lying down, dynamic standing, sitting, walking, and running, more variations of these activities exist and are likely adopted in a real life setting. In this regard, it is important to note that this study initially included stair ascending and descending as well as biking. Because the preliminary validation of the performance of the classification model obtained with biaxial accelerometers in CE gave us a low sensitivity for these activities (*i*.*e*., 37% for climbing stairs and 74% for biking), they were not included in the classification model nor were they further investigated. Firstly, the protocol used to measure stairs climbing included 2 min of ascending and descending stairs. Since both patterns are different, the method used was not specific enough for a good classification. Secondly, the sampling rate of 5 s for the accelerations and 1 min for the step count was not high enough to measure biking. It could be hypothesized that a higher time spent doing the activity and a higher sampling frequency would have been helpful in this case. The decision to maintain the sampling frequency was mostly informed by the fact that a higher sampling frequency would have overwhelmed the storage capacities of the devices over longer sampling periods under real life conditions. Finally, the use of an another accelerometer and/or a GPS could have help to measure biking [25].

The results of the present study highlight the high accuracy and reproducibility of both classification models in CE and UE. To the best of our knowledge, no study has investigated and validated several activities under unrestrictive conditions for a period longer than 24 hours. The main reasons that explain this is that the internal memory capacity of sensors is limited and quickly fills up when data are sampled at high frequency. In this study we show that our model, which was developed while using a lower frequency of sampling, has comparable validity to previously published work as far as activity recognition is concerned with the major advantage of being useful for the measurement of several activities that make up for a great proportion of daily life over a much longer duration (up to 7 days). This study also shows that activity recognition models including either 6 or 8 features (i.e. Biaxial vs. Triaxial accelerometers, respectively) are not different in terms of their performance, at least when investigating the five categories of activities presented in this paper. Future research in this area is needed to develop classification models that are more sensitive to capture activities such as biking, stair-climbing as well as transitions from one activity to another.

## Conclusions

The classification model developed in this study was shown to be accurate and reliable over 24 hours in UE. Our results show no significant benefit of using eight compared to six features to determine the time spent performing five activities as far as the present classification model is concerned. The study highlights the potential use of this classification model in applied research aimed at investigating the time spent performing activities.

## References

[1] Preece SJ, Goulermas JY, Kenney LPJ, Howard D, Meijer K, Crompton R. Activity identification using body-mounted sensors-a review of classification techniques. Physiol Meas. 2009;30(4):R1–R33. 10.1088/0967-3334/30/4/R01 . 19342767

[2] Yang CC, Hsu YL. A review of accelerometry-based wearable motion detectors for physical activity monitoring. Sensors (Basel). 2010;10(8):7772–88. Epub 2010/01/01. 10.3390/s100807772 sensors-10-07772 [pii]. 22163626 PMC3231187

[3] Fahrenberg J, Foerster F, Smeja M, Muller W. Assessment of posture and motion by multichannel piezoresistive accelerometer recordings. Psychophysiology. 1997;34(5):607–12. Epub 1997/09/23. . 9299915 10.1111/j.1469-8986.1997.tb01747.x

[4] Karantonis DM, Narayanan MR, Mathie M, Lovell NH, Celler BG. Implementation of a real-time human movement classifier using a triaxial accelerometer for ambulatory monitoring. IEEE Trans Inf Technol Biomed. 2006;10(1):156–67. Epub 2006/02/01. . 16445260 10.1109/titb.2005.856864

[5] Van Laerhoven K, Cakmakci O. What shall we teach our pants? IEEE. 2000:77–83.

[6] Veltink PH, Bussmann HB, de Vries W, Martens WL, Van Lummel RC. Detection of static and dynamic activities using uniaxial accelerometers. IEEE Trans Rehabil Eng. 1996;4(4):375–85. Epub 1996/12/01. . 8973963 10.1109/86.547939

[7] Bao L, Intille S. Activity recognition from user-annotated acceleration data. Pervasive Computing. 2004:1–17.

[8] Parkka J, Ermes M, Korpipaa P, Mantyjarvi J, Peltola J, Korhonen I. Activity classification using realistic data from wearable sensors. IEEE Trans Inf Technol Biomed. 2006;10(1):119–28. Epub 2006/02/01. . 16445257 10.1109/titb.2005.856863

[9] Foerster F, Smeja M., Fahrenberg J. Detection of posture and motion by accelerometry: A validation study in ambulatory monitoring. Biomed Instrum Technol. 1999;15:571–83.

[10] Lyons GM, Culhane KM, Hilton D, Grace PA, Lyons D. A description of an accelerometer-based mobility monitoring technique. Med Eng Phys. 2005;27(6):497–504. Epub 2005/07/02. doi: S1350-4533(04)00219-X [pii] 10.1016/j.medengphy.2004.11.006 . 15990066

[11] Uiterwaal M, Glerum EB, Busser HJ, van Lummel RC. Ambulatory monitoring of physical activity in working situations, a validation study. J Med Eng Technol. 1998;22(4):168–72. Epub 1998/07/29. . 9680600 10.3109/03091909809032535

[12] van den Berg-Emons HJG, Bussmann JBJ, Balk AHMM, Stam HJ. Validity of ambulatory accelerometry to quantify physical activity in heart failure. Scand J Rehabil Med. 2000;32(4):187–92. . 11201626 10.1080/003655000750060940

[13] Busser HJ, Ott J, van Lummel RC, Uiterwaal M, Blank R. Ambulatory monitoring of children's activity. Med Eng Phys. 1997;19(5):440–5. Epub 1997/07/01. doi: S1350-4533(97)00007-6 [pii]. . 9338884 10.1016/s1350-4533(97)00007-6

[14] Ermes M, Parkka J, Mantyjarvi J, Korhonen I. Detection of daily activities and sports with wearable sensors in controlled and uncontrolled conditions. IEEE Trans Inf Technol Biomed. 2008;12(1):20–6. Epub 2008/02/14. 10.1109/TITB.2007.899496 . 18270033

[15] Long X, Yin B, Aarts RM. Single-accelerometer-based daily physical activity classification. Conf Proc IEEE Eng Med Biol Soc. 2009;2009:6107–10. Epub 2009/12/08. 10.1109/IEMBS.2009.5334925 . 19965261

[16] St-Onge M, Mignault D, Allison DB, Rabasa-Lhoret R. Evaluation of a portable device to measure daily energy expenditure in free-living adults. The American journal of clinical nutrition. 2007;85(3):742–9. Epub 2007/03/09. doi: 85/3/742 [pii]. . 17344495 10.1093/ajcn/85.3.742

[17] Abeel T, de Peer YV, Saeys Y. Java-ML: A Machine Learning Library. Journal of Machine Learning Research. 2009;10:931–4.

[18] Bonomi AG, Plasqui G, Goris AH, Westerterp KR. Improving assessment of daily energy expenditure by identifying types of physical activity with a single accelerometer. J Appl Physiol. 2009;107(3):655–61. Epub 2009/06/27. doi: 00150.2009 [pii] 10.1152/japplphysiol.00150.2009 . 19556460

[19] Banerjee MC, McSweeney M, Sinha L. Debajyoti Beyond Kappa: A Review of Interrater Agreement Measures. The Canadian Journal of Statistics / La Revue Canadienne de Statistique 1999;27(1):3–23.

[20] Bonomi AG, Goris AH, Yin B, Westerterp KR. Detection of type, duration, and intensity of physical activity using an accelerometer. Medicine and science in sports and exercise. 2009;41(9):1770–7. Epub 2009/08/07. 10.1249/MSS.0b013e3181a24536 . 19657292

[21] Zhang K, Werner P, Sun M, Pi-Sunyer FX, Boozer CN. Measurement of human daily physical activity. Obesity research. 2003;11(1):33–40. . 12529483 10.1038/oby.2003.7

[22] Mathie MJ, Celler BG, Lovell NH, Coster AC. Classification of basic daily movements using a triaxial accelerometer. Med Biol Eng Comput. 2004;42(5):679–87. Epub 2004/10/27. . 15503970 10.1007/BF02347551

[23] Bonomi AG, Westerterp KR. Advances in physical activity monitoring and lifestyle interventions in obesity: a review. International journal of obesity (2005). 2012;36(2):167–77. 10.1038/ijo.2011.99 . 21587199

[24] Dunstan DW, Howard B, Healy GN, Owen N. Too much sitting—a health hazard. Diabetes research and clinical practice. 2012;97(3):368–76. 10.1016/j.diabres.2012.05.020 . 22682948

[25] Duncan MJ, Badland HM, Mummery WK. Applying GPS to enhance understanding of transport-related physical activity. J Sci Med Sport. 2009;12(5):549–56. Epub 2009/02/25. doi: S1440-2440(08)00210-7 [pii] 10.1016/j.jsams.2008.10.010 . 19237315

